# Integrated multi-omics analysis reveals that Gongying San ameliorates subclinical mastitis by modulating intestinal microbiota and metabolites in dairy cows

**DOI:** 10.3389/fvets.2025.1589900

**Published:** 2025-06-09

**Authors:** Guoqing Zhao, Hongxia Li, Liwei Huang, Yu Cheng, Jia Liu, Ruigao Song, Xi Wang

**Affiliations:** ^1^College of Animal Science, Shanxi Agricultural University, Taigu, China; ^2^Department of Obstetrics and Gynecology, Reproductive Medicine Center, Shanxi Bethune Hospital, Shanxi Academy of Medical Sciences, Third Hospital of Shanxi Medical University, Tongji Shanxi Hospital, Taiyuan, China

**Keywords:** dairy cows, subclinical mastitis, Gongying San, intestinal microbiota, metabonomics

## Abstract

**Introduction:**

Subclinical mastitis (SCM) is a common disease in dairy cows associated with dysbiosis of the gastrointestinal microbiota and systemic inflammatory response. Gongying San (GYS), a commonly used herbal formula for the treatment of mastitis, has anti-inflammatory, antibacterial and antioxidant effects, but the underlying mechanisms remain unknown. Therefore, we performed a multi-omics analysis to determine the effects of GYS on intestinal microbiota and metabolites in cows with SCM.

**Methods:**

A total of 32 Holstein cows were divided into four groups of 8 cows each, including healthy control group, subclinical mastitis group, GYS treatment group (290 g/day) and ceftiofur treatment group (2.2 mg/kg bw).

**Results:**

GYS significantly increased milk yield, lactose and milk protein, and decreased somatic cell count (SCC) in milk from cows with SCM. In the serum, GYS decreased the levels of lipopolysaccharide (LPS), interleukin-2 (IL-2), interleukin-4 (IL-4), interleukin-8 (IL-8), interleukin-10 (IL-10), tumor necrosis factor-α (TNF-α) and malondialdehyde (MDA) and increased the concentration of superoxide dismutase (SOD). In addition, there was an increase in *UCG-010* and *Blautia* and a decrease in *Bacteroides, Lachnospiraceae*, and *Agathobacter* in feces after GYS treatment. Fecal untargeted metabolomics showed that GYS supplementation mainly downregulated inflammation-related metabolism, including arachidonic acid and choline metabolism.

**Discussion:**

In the treatment of SCM, GYS showed multi-target therapeutic advantages of anti-inflammatory, antioxidant and immunomodulatory properties compared to antibiotics. *Bacteroides, Lachnospiraceae* and *UCG-010* may be involved in the regulation of inflammation through 3-oxo-Δ4bile acids and phosphatidylcholine.

## 1 Introduction

Mastitis represents a significant challenge to the advancement of the global dairy industry and exerts a considerable influence on the economic viability of animal husbandry. In addition to impairing the quality and yield of milk, the disease also endangers the well-being and health of dairy cows (1). In particular, SCM has a high incidence in dairy cows due to its hidden nature and long incubation period, accounting for approximately 90–95% of mastitis cases (2). The total economic cost of dairy cattle diseases is estimated to be $65 billion per year globally, of which SCM alone contributes about $9 billion (3). SCM reduces milk production by 5.6–17.2% during lactation, with a loss of $38 per cow per lactation due to SCM, and up to $66.6 per cow on urban farms (4). However, the current therapeutic regimen faces serious challenges in terms of antibiotic dependence, the spread of resistance and the control of recurrence rates. Therefore, there is a need to find a more efficient treatment option that takes into account the pathogenesis of mastitis.

In examining the etiology of mastitis, researchers have come to recognize that the disease is not solely a consequence of a localized inflammatory response. Rather, it may also be influenced by alterations in the structure of the host’s microbial community. Some studies have demonstrated that the fecal microbiota of cows with mastitis can be transferred to germ-free mice, which then exhibit mastitis and serum inflammation [increased TNF-α, interleukin-17 (IL-17), LPS levels] (5). This evidence suggests that there may be a “gastroenterogenic mastitis” pathogenic pathway for the development of mastitis (6). The concept of the “intestinal-mammary axis” has also been proposed by some scientists (7). It can be surmised that intestinal microbiota and their metabolites may migrate to the mammary gland via the bloodstream, thereby modulating mastitis. Currently, antibiotics remain the primary choice of medication for clinical treatment (8). However, the indiscriminate use of antibiotics not only increases production costs, promotes the development of drug-resistant pathogens, and disrupts the intestinal microbiota, but also poses a significant threat to public health (9). With growing concerns about antibiotic dependency, exploring alternatives to antibiotics has become a key area in addressing mastitis. Therefore, we attempted to improve the intestinal microbiota structure through nutritional modulation in order to alleviate SCM in dairy cows.

Herbal medicine represents a promising avenue for the treatment of mastitis. The use of herbal combination therapy offers a practical approach to reducing inflammation and regulating intestinal microbiota (10). Taraxacum is a member of the Asteraceae family and contains a variety of phytochemicals that are beneficial to animal health. These include phenolic compounds, sesquiterpene lactones, polysaccharides, and flavonoids (11). It has been demonstrated that the administration of taraxacum to mid-lactation cows can enhance lactation performance and stimulate amino acid metabolism and rumen fermentation (12, 13). Taraxacum extract has been demonstrated to alleviate LPS-induced oxidative stress by scavenging cellular reactive oxygen species (ROS) and increasing antioxidant enzyme activities. Furthermore, it has been shown to activate the nuclear factor erythroid 2-related factor 2 (Nrf2) signaling pathway, which in turn promotes the expression of antioxidant genes (14). Additionally, the extract has been observed to significantly inhibit the expression of TNF-α and intercellular cell adhesion molecule-1 (ICAM-1), thereby alleviating inflammation (15). The anti-inflammatory effects of lonicerae japonicae flos have been shown to down-regulate the release of IL-1β, IL-6, and TNF-α (16). *Forsythia suspensa* is commonly used in the treatment of various inflammatory diseases, for example it significantly reduces the elevated TNF-α and IL-6 caused by colitis and improves colonic tissue damage (17). Gongying San is a classic herbal formula consisting of taraxacum, lonicerae japonicae flos, forsythia suspensa, retinervus luffae fructus, bulbus fritillariae thunbergii, hibisci mutabilis folium and tetrapanax papyrifer. In Gongying San, the combination of dandelion with other herbs can further enhance its efficacy through the ‘multi-component multi-target multi-pathway’ mode of action, which makes it more promising in the treatment of various inflammatory and infectious diseases. Network pharmacology and molecular-docking analyses revealed that quercetin, lignans and kaempferol were the main active components of Gongying San in the treatment of mastitis, and TNF, IL-6, IL1β, ICAM1, and CXCL8 were its key targets (18). However, there is a lack of standardized clinical validation of Gongying San. Accordingly, the objective of this study was to examine the impact of Gongying San, a Chinese herbal compound, on intestinal microbiota and their metabolites, lactation performance, and inflammatory response in cows with SCM.

## 2 Materials and methods

### 2.1 Animal, diets, and experiment design

**Figure 1:**
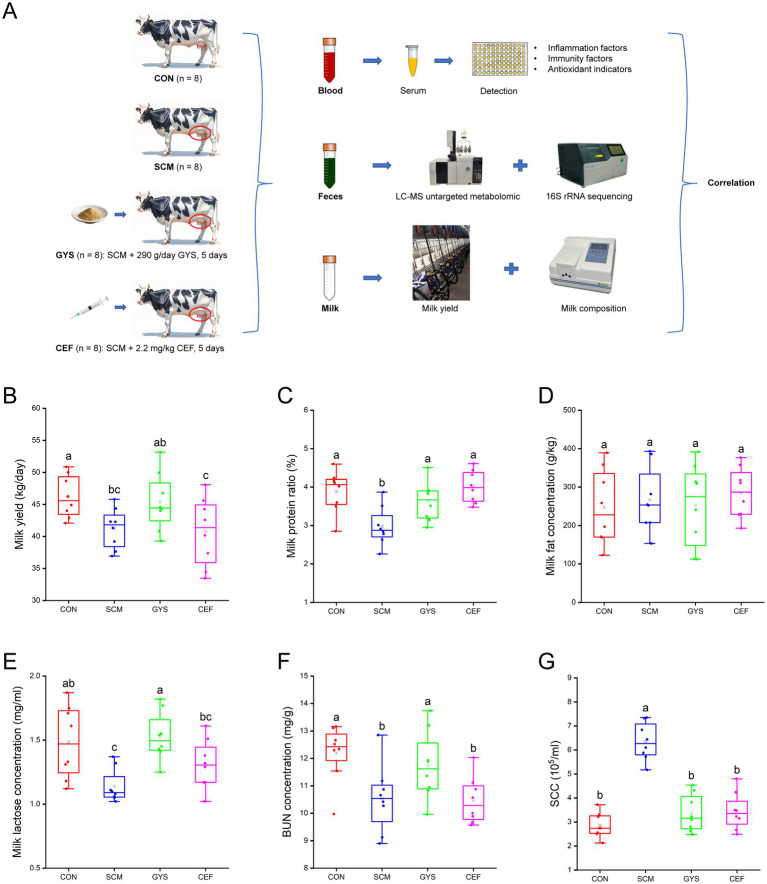
Experimental design and lactation performance of dairy cows. **(A)** Experimental design. **(B)** Milk yield. **(C)** Milk protein. **(D)** Milk fat. **(E)** Milk lactose. **(F)** BUN, blood urea nitrogen. **(G)** SCC, somatic cell counts. CON, control group; SCM, subclinical mastitis group; GYS, Gongying San group, the Gongying San addition level was 290 g/day per cow; CEF, antibiotic group, antibiotic treatment consisted of daily intramuscular injections of ceftiofur hydrochloride (2.2 mg/kg bw) into the neck region for 5 days. Differences in letters on the box-and-line plot indicate significant differences between groups (*p* < 0.05). ^a, b, c^ Different letters differed significantly (*p* < 0.05).

The experimental design has been shown in Figure 1. This study used 32 Holstein dairy cows in mid-lactation (body weight 655 ± 18.7 kg, days to lactation 110 ± 12.4 d) reared at ZiLin Ranch of the Shanxi, China. These cows have not been treated with antibiotics or other drugs within the past year and have been raised in the same environment. The ingredients and chemical composition of basal diet are shown in Supplementary Table S1. SCM was diagnosed by the result of California mastitis test (CMT) was weakly positive (600,000 < SCC < 1,000,000 cells/mL) and no clinical symptoms in udders (19). The experimental design has been shown in Figure 1. Eight healthy cows were used as control group (CON; *n* = 8) (SCC < 100, 000cells/mL; no clinical symptoms in udders; CMT results were negative), Twenty-four cows with SCM were selected from the farm and randomly divided into the subclinical mastitis group (SCM; *n* = 8); Gongying San group (GYS; *n* = 8); ceftiofur hydrochloride group (CEF; *n* = 8). In GYS group, the Gongying San (New Century Pharmaceutical Co., Ltd., Hebei, China) was taken through the mouth 290 g/day per cow for 5 days; the detailed composition of GYS is listed in Table 1. In CEF group, antibiotic treatment consisted of daily intramuscular injections of ceftiofur hydrochloride (2.2 mg/kg bw) into the neck region for 5 days. Two weeks before the start of the experiment, the four groups of cows were kept in four different pens and fed the basal diet. The drug treatment started on the first day of the experiment and was carried out for five consecutive days. Milk, serum and fecal samples were collected on the sixth day. The drug treatment started on the first day of the experiment and was carried out for five consecutive days. Milk, serum and fecal samples were collected on the sixth day. This was followed by serum index tests, milk yield tests, milk composition tests, 16SrRNA sequencing and non-targeted metabolomics analyses, and finally correlation analyses.

**Table 1:** The composition of Gongying San.

Chinese name	Latin name	Content (g)
Pu Gong Ying	Taraxacum mongolicum Hand. -Mazz.	60
Jin Yin Hua	Lonicerae Japonicae Flos	60
Lian Qiao	Forsythia suspensa	60
Si Gua Luo	Retinervus Luffae Fructus	30
Zhe Bei Mu	Bulbus Fritillariae Thunbergii	30
Fu Rong Ye	Hibisci Mutabilis Folium	25
Tong Cao	Tetrapanax papyrifer	25

### 2.2 Milk sampling and analysis

Cows were milked three times a day at 07: 30, 13:30, and 19:30, utilizing an automated milking system. The data pertaining to the yield of milk were recorded on a daily basis by a dairy herd management software program. Milk samples from each cow were collected 7:00, 13:00, and 19:00 and mixed with a ratio of 4:3:3 on the sixth day of the experiment. The mixed milk samples from each cow were collected into a 15-ml sterile centrifuge tubes and stored at 4°C. The milk protein was determined by Kjeldahl method. The milk fat was determined by Soxhlet extractor method. The concentration of milk lactose was analyzed using a milk lactose test kit (Sangon Biotech Co., Ltd., Shanghai, China). The milk SCC was determined by Microscopic method. The concentration of milk urea nitrogen (MUN) was analyzed using a MUN test kit (Sangon Biotech Co., Ltd., Shanghai, China).

### 2.3 Serum collection and analysis

Blood samples were collected through the tail vein at 06: 30, on the sixth day of the experiment. One tube of blood sample was taken from each cow. Blood samples were allowed to stand for 30 min at room temperature and then centrifuged at 3,000 × g for 15 min at 4°C to separate the serum. The collected serum samples were stored at - 80°C for biochemical analysis. Blood urea nitrogen (BUN) was analyzed using a BUN test kit (Sangon Biotech Co., Ltd., Shanghai, China). The concentrations of total protein (TP) and albumin (ALB) were analyzed by a TP colorimetric test kit (BCA method) (Sangon Biotech Co., Ltd., Shanghai, China) and an ALB assay kit (bromocresol green colorimetry) (Sangon Biotech Co., Ltd., Shanghai, China), respectively. IL-2, IL-4, IL-6, IL-8, IL-10, TNF-α, prostaglandin E2 (PGE2), glutathione peroxidase (GSH-Px), SOD, MDA, and LPS concentrations were determined by the corresponding bovine enzyme-linked immunosorbent assay (ELISA) kits (Sangon Biotech Co., Ltd., Shanghai, China). The concentrations of IgA, IgG, and IgM were measured by bovine immunoglobulin ELISA kits (Sangon Biotech Co., Ltd., Shanghai, China).

### 2.4 Feces sample collection

Feces samples were collected from the rectum at 09:00, on the sixth day of the experiment. Fecal samples from each cow were stored in two sterile 5 mL tubes at - 80°C for microbiological and metabolite analysis.

### 2.5 DNA extraction, sequencing and data processing

Total community genomic DNA extraction was performed using a E. Z. N. A^™^ Mag-Bind Soil DNA Kit (#M5635-02, Omega, United States), following the manufacturer’s instructions. We measured the concentration of the DNA using a Qubit4.0 (Thermo, United States) to ensure that adequate amounts of high-quality genomic DNA had been extracted.

The objective of this study was to target the V3-V4 hypervariable region of the bacterial 16S rRNA gene. Polymerase chain reaction (PCR) was initiated immediately following the extraction of DNA. The 16S rRNA V3-V4 amplicon was amplified using 2 × Hieff^®^ Robust PCR Master Mix (#10105ES03, Yeasen, China). Two universal bacterial 16S rRNA gene amplicon PCR primers (PAGE-purified) were utilized: the amplicon PCR forward primer (CCTACGGGNGGCWGCAG) and the amplicon PCR reverse primer (GACTACHVGGGTATCTAATCC). The reaction was configured as follows: microbial DNA (10 ng/μl) (2 μL); amplicon PCR forward primer (10 μM) (1 μL); amplicon PCR reverse primer (10 μM) (1 μL); 2 × Hieff^®^ Robust PCR Master Mix (30 μL). The plate was then sealed and subjected to PCR in a thermal instrument (Applied Biosystems 9700, United States) using the following program: Initially, the program subjected the samples to a cycle of denaturing at 95°C for 3 min. This was followed by five cycles of denaturing at 95°C for 30 s, annealing at 45°C for 30 s, and elongation at 72°C for 30 s. The subsequent twenty cycles consisted of denaturing at 95°C for 30 s, annealing at 55°C for 30 s, and elongation at 72°C for 30 s. The program culminated with a final extension at 72°C for 5 min. The amplification products were subjected to electrophoresis in 2% (w/v) agarose gels in TBE buffer (Tris, boric acid, EDTA) containing ethidium bromide (EB) and were then visualized under UV light.

We used Hieff NGS™DNA Selection Beads (#10105ES03, Yeasen, China) to purify the free primers and primer dimer species in the amplicon product. Samples were delivered to Sangon BioTech for library construction using universal Illumina adaptor and index. Before sequencing, the DNA concentration of each PCR product was determined using a Qubit^®^4.0Green double-stranded DNA assay and it was quality controlled using a bioanalyzer (Agilent2100, United States). Depending on coverage needs, all libraries can be pooled for one run. The amplicons from each reaction mixture were pooled in equimolar ratios based on their concentration. Sequencing was performed using the Illumina MiSeq system (Illumina MiSeq, United States), according to the manufacturer’s instructions.

The primer junction sequences must be removed using Cutadapt (version 1.18^1^), after which the PEAR software (version 0.9.8^2^) will be used to merge pairs of reads into a single sequence based on the overlap relationship between PE reads. PRINSEQ (version 0.20.4^3^) will then be used to filter the quality of each sample data for quality control purposes. The operational taxonomic units (OTU) clustering of non-repetitive sequences was conducted according to 97% similarity using Usearch (version 11.0.667^4^). The taxonomic analysis of OTU representative sequences was conducted in accordance with the Silva 16S rRNA database (version 138.2^5^), utilizing the RDP classifier (version 2.12^6^). The calculation of the alpha diversity index (Chao1 and Shannon) is performed using the Mothur software (version 1.43.0^7^). The distance algorithms of the principal coordinates analysis (PCoA) were weighted normalized UniFrac and unweighted UniFrac, which were performed by using the vegan package in R (version 3.6.0^8^). Linear discriminant analysis effect size (LEfSe) was performed using LEfSe software (version 1.1.0^9^) to discover the differential microbiota between groups and the extent to which the differential microbiota influenced the differences between groups was expressed by Linear discriminant analysis (LDA). Significantly different microbiotas were defined as LDA > 3.5 and *p* < 0.05 (Student’s t-test), the FDR method was applied to adjust *p*-values.

### 2.6 Feces metabolomics analysis

After thawing the samples on ice, 20 mg (±1 mg) of feces from each cow was mixed with 400 μL of 70% methanol–water internal standard extract and vortexed for 3 min. The samples were ultrasonicated for 10 min in an ice-water bath, removed from the samples, vortexed for 1 min, and allowed to stand for 30 min in a refrigerator at −20°C. The supernatant was extracted by centrifugation at 12,000 r/min for 10 min at 4°C, and then centrifuged again at 12,000 r/min for 3 min at 4°C. 200 μL of supernatant was extracted for LC–MS analysis. Chromatographic separation was performed on a HSS T3 chromatographic column (2.1 mm × 100 mm, 1.8 μm, Waters). Mobile phase A contained 0.1% formic acid and water, and mobile phase B contained 0.1% formic acid and acetonitrile at a flow rate of 0.4 mL/min, with an instrumental column temperature of 40°C and a sample volume of 4 μL. Separation gradient: 95%: 5% at 0 min; 80%: 20% at 2 min; 40%: 60% at 5 min; 1%: 99% for 6–7.5 min; 95%: 5% for 7.6–10 min. Data were collected in positive and negative ion modes with a duration of 10 min for ESI + and ESI-, ion spray voltage of 5,000 and −4,000 V and temperature of 550 and 450°C.

Mass spectrum peaks were extracted, aligned, and retention time corrected from LC–MS raw data using the xcms package in R software. Peaks with > 50% missing in each set of samples were filtered, blanks were filled, and peak areas were corrected. The filtered peaks were used for metabolite identification by searching Human metabolome database (HMDB^10^). Principal component analysis (PCA) analysis was performed using the prcomp function in the R software, and Orthogonal projections to latent structures-discriminate analysis (OPLS-DA) analysis was performed using the MetaboAnalystR package OPLSR. Anal function in the R software. The criteria for determining differential metabolites were variable importance in projection (VIP) > 1 and *p* < 0.05 (Student’s *t*-test), the FDR method was applied to adjust *p* values. The metabolite content data were processed using unit variance scaling (UV), and all samples were analyzed by cluster analysis. Annotation of metabolic pathways using the KEGG database.^11^ Spearman’s correlation analysis was executed by employing the Scipy packages of Python (version 1.26.4), and the R heatmap package was utilized for the purpose of visualization.

### 2.7 Statistical analysis

A comprehensive analysis of lactation performance, serum markers, microorganisms, and metabolites were conducted using one-way analysis of variance (ANOVA) and Student’s *t*-test, with SPSS software (version 22.0) serving as the statistical analysis tool. Differences were statistically significant when *p* < 0.05.

## 3 Results

### 3.1 Effect of GYS treatment on milk yield and composition

As shown in Figures 1B,C,E,F, compared with the CON group, SCM group significantly decreased milk yield (*p =* 0.024), milk protein (*p =* 0.001), milk lactose (*p =* 0.002), BUN (*p =* 0.005) and significantly increased SCC (*p* < 0.001). However, there was a significant recovery of milk protein (*p =* 0.014), milk lactose (*p* < 0.001), BUN (*p =* 0.039) and SCC (*p* < 0.001) and a trend toward higher Milk yield (*p =* 0.058) after GYS treatment compared to the SCM group. Milk fat content did not differ significantly among the four groups (Figure 1D). Although CEF significantly reduced SCC (*p* < 0.001), it did not increase milk yield (Figures 1B,G).

### 3.2 Effect of GYS treatment on blood biochemical indexes

As shown in Table 2, TP, ALB and GLB did not change significantly in the four groups. In terms of inflammation, IL-2 (*p =* 0.013), IL-4 (*p =* 0.019), IL-6 (*p =* 0.028), IL-8 (*p =* 0.011), IL-10 (*p =* 0.016), and TNF-α (*p =* 0.018) levels were significantly higher in the SCM group compared to the CON group, with IL-2 (*p =* 0.023), IL-4 (*p =* 0.004), IL-8 (*p =* 0.041), IL-10 (*p =* 0.008), and TNF-α (*p =* 0.016) levels being significantly reduced after GYS treatment, while IL-4 (*p =* 0.045) and TNF-α (*p =* 0.009) levels were reduced by CEF treatment. In terms of immunity, SCM significantly increased IgA (*p =* 0.015) levels compared to the CON group, whereas there was a significant decrease in IgA (*p =* 0.009) levels after GYS treatment. In terms of antioxidants, SOD (*p =* 0.019) levels were significantly lower in the SCM group compared to the CON group, significantly higher in GYS and CEF groups (*p* < 0.050). MDA (*p =* 0.002) levels were significantly higher in the SCM group compared to the CON group, significantly lower in the GYS and CEF groups (*p* < 0.050).

**Table 2:** Effect of GYS on the inflammatory cytokine, oxidative stress indexes and LPS in serum of dairy cows with SCM.

Items	Groups (*n* = 4)				SEM	*P*-value
	CON	SCM	GYS	CEF		
TP, mg/ml	2.01	1.99	2.04	2.01	0.05	0.993
ALB, mg/ml	1.33	1.33	1.34	1.32	0.04	0.997
GLB, μg/ml	697	686	683	686	16.0	0.991
IgA, μg/ml	123^b^	153^a^	120^b^	136^ab^	4.56	0.034
IgG, mg/ml	2.45	2.55	2.39	2.53	0.05	0.731
IgM, μg/ml	134	138	143	143	2.66	0.705
IL-2, ng/liter	309^b^	383^a^	316^b^	361^ab^	10.9	0.036
IL-4, ng/liter	76.5^b^	96.5^a^	71.2^b^	79.7^b^	3.18	0.023
IL-6, ng/liter	21.2	23.5	21.5	22.1	0.37	0.124
IL-8, ng/liter	510^b^	595^a^	529^b^	552^ab^	11.8	0.059
IL-10, ng/liter	34.2^b^	43.4^a^	33.1^b^	43.6^a^	1.49	0.006
TNF-α, ng/liter	217^b^	270^a^	217^b^	211^b^	8.23	0.029
PGE_2_, ng/liter	403	465	426	382	16.6	0.337
GSH-Px, ng/liter	712	647	690	704	22.0	0.742
SOD, pg./ml	409^a^	339^b^	423^a^	414^a^	10.9	0.031
MDA, nmol/ml	16.9^b^	21.5^a^	17.4^b^	18.5^b^	0.54	0.006
LPS, ng/liter	475^ab^	552^a^	427^b^	533^ab^	19.1	0.078

TP, total protein; ALB, albumin; GLB, globulin; IL, interleukin; TNF-α, tumor necrosis factor-α; PGE2, prostaglandin E2; GSH-Px, glutathione peroxidase; MDA, malondialdehyde; SOD, superoxide dismutase; LPS, lipopolysaccharide; SEM, standard error of the mean; Con, control group; SCM, subclinical mastitis group; GYS, Gongying San group, the Gongying San addition level was 290 g/day per cow; CEF, antibiotic group, antibiotic treatment consisted of daily intramuscular injections of ceftiofur hydrochloride (2.2 mg/kg bw) into the neck region for 5 days. ^a, b^ = within a row, different letters differed significantly (*P* < 0.05).

### 3.3 Differences in diversity, richness and composition of fecal microbiota

**Figure 2:**
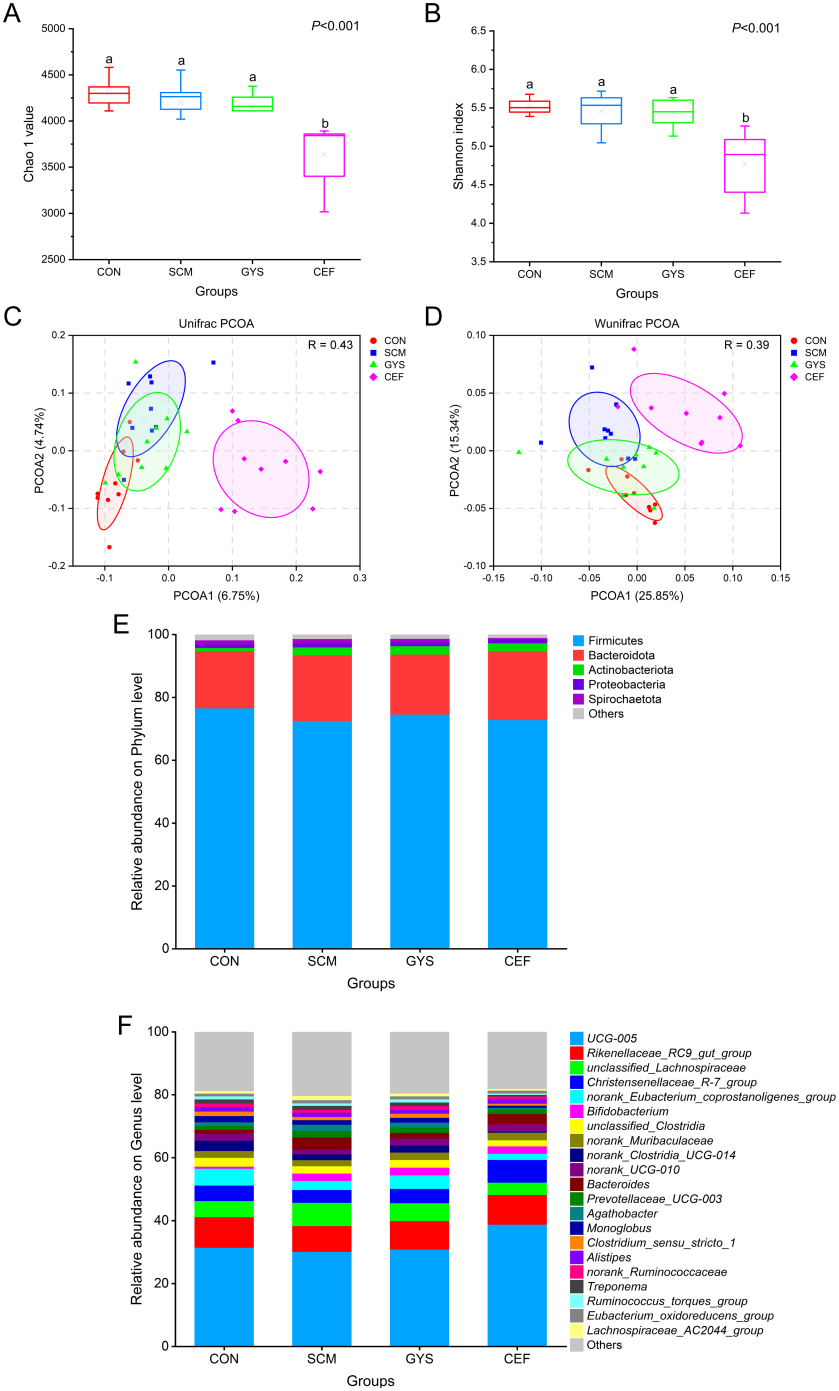
Changes in α-and β-diversity as well as feces bacterial community of dairy cows. Chao1 **(A)** and Shannon **(B)** index (α-diversity) reflect the diversity and richness of fecal microbiota. ^a,b^Different letters differed significantly (*p* < 0.05). Unweighted unifrac distance **(C)** and weighted unifrac distance **(D)** (β-diversity), which showed the profile of fecal microbial communities among groups. Relative abundance of phylum **(E)** and genus **(F)** between four groups.

The effect of GYS on intestinal microbiota in dairy cows was determined by 16S rRNA high-throughput sequencing. As the number of reads sampled increases, the alpha diversity index gradually reaches a plateau, indicating that the amount of data for this sequencing is sufficient (Supplementary Figure S1). The results of the alpha diversity analysis indicated that there were no significant differences in the chao1 index and shannon index between the CON, SCM, and GYS groups. However, a significant decrease was observed in the CEF (*p* < 0.001) group (Figures 2A,B). In Figures 2C,D, Regarding beta diversity, PCOA of intestinal microbiota by unifrac and wunifrac algorithms. An analysis of similarity (Anosim) was used to test whether the differences between groups were significantly greater than the differences within groups, with R > 0 indicating that the groupings were meaningful. Significant differences in microbial composition were observed in the GYS group compared to the SCM group (*p =* 0.048), but similar to the CON group (*p =* 0.118). Also the microbial composition of the CEF group was significantly different from the other three groups (*p* < 0.001). At the phylum level, Firmicutes, Bacteroidota, Actinobacteriota, Proteobacteria, and Spirochaetota were the dominant groups in four groups (Figure 2E). At the genus level, the most prevalent intestinal microbiota include *UCG-005*, *Rikenellaceae_RC9_intestinal_group*, *unclassified_Lachnospiraceae*, Christensenellaceae_R_7_group, norank_Eubacterium_coprostanoligenes_group, *Bifidobacterium*, *unclassified_Clostridia*, and *norank_Muribaculaceae* (Figure 2F).

### 3.4 Effect of GYS on fecal microbiota content

**Figure 3:**
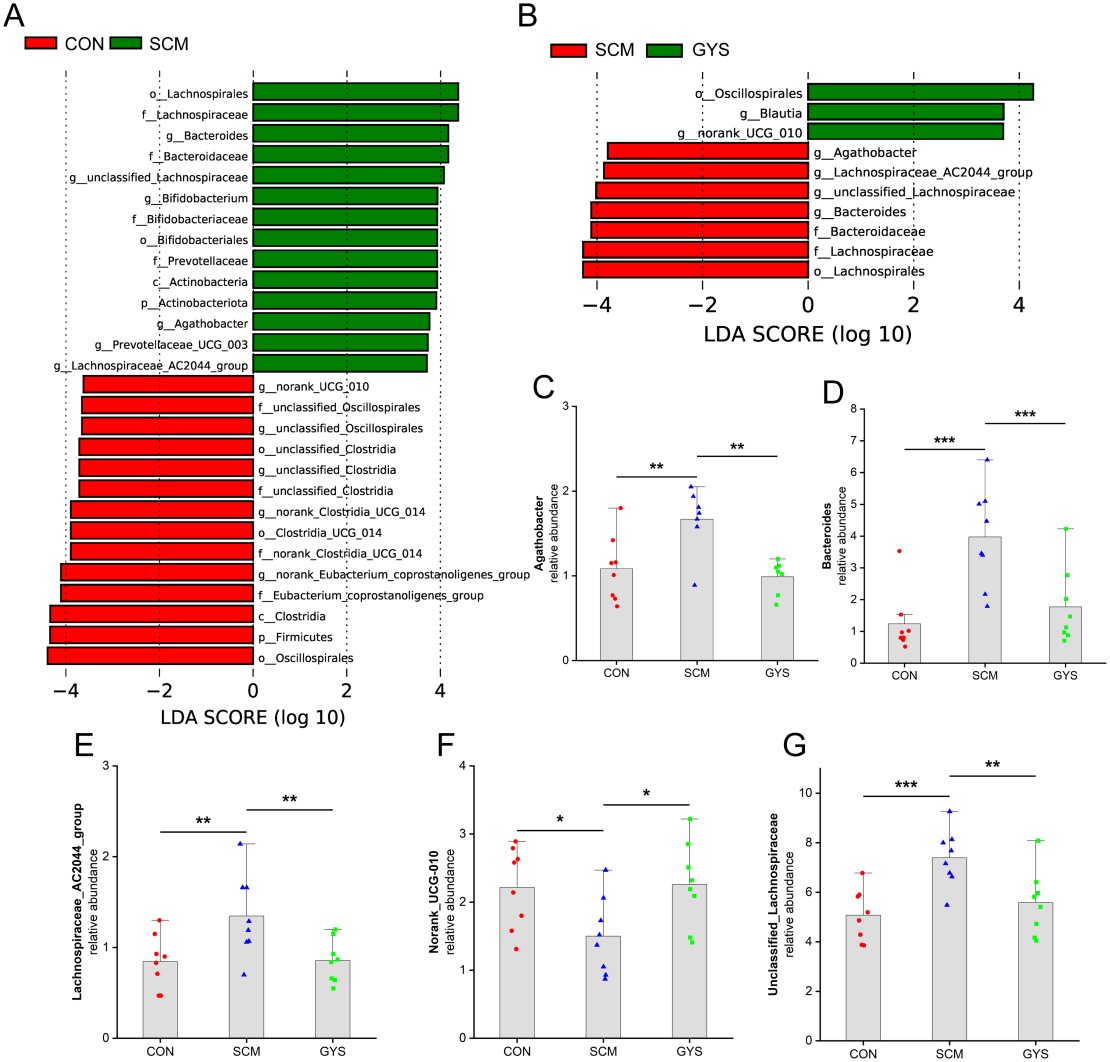
Composition of the feces bacterial community of dairy cows. **(A,B)** Bar chart of linear discriminant analysis (LDA) score of different bacterial taxa in the feces, respectively. LDA score > 3.5. **(C–G)** Bar charts showing the relative abundance of common differential microbiota in CON versus SCM and SCM versus GYS dairy cow fecal samples. *p < 0.05, **p < 0.01, ***p < 0.001.

To assess how GYS administration affected the dairy cow intestinal microbial composition, linear discriminant analysis effect size (LEfSe) analysis was utilized to analyse the bacterial microbiota. We found significant differences in the relative abundance of 10 genera in the comparison of the CON group with the SCM group, while 6 genera were found to be significantly different in the comparison of the SCM group with the GYS group, with 5 genera being common to the colony (Figures 3A,B). Compared with CON, the relative abundances of *unclassified_Lachnospiraceae* (*p* < 0.001), *Agathobacter* (*p =* 0.003), *Bacteroides* (*p* < 0.001) and *Lachnospiraceae_AC2044_group* (*p =* 0.007) in the SCM were increased and decreased in the GYS group (*p* < 0.01). The relative abundances of *norank_UCG-010* (*p =* 0.026) were lower in the CFE than in the CON and increased in the GYS group (*p =* 0.019) (Figures 3C–G).

### 3.5 Differences in fecal metabolites

**Figure 4:**
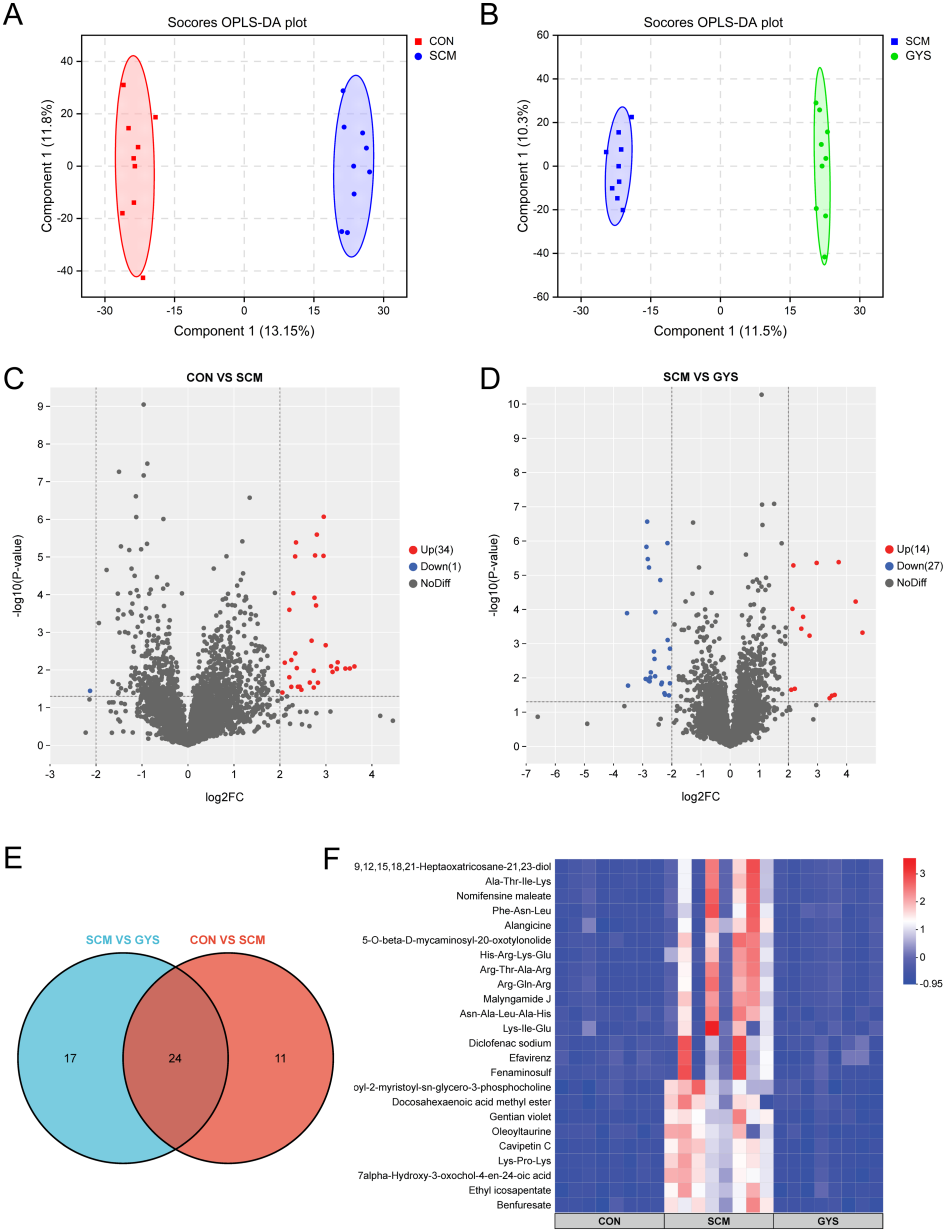
Fecal metabolome of CON, SCM and CFE cows. Scores plot of orthogonal partial least square discriminant analysis (OPLS-DA) for CON versus SCM **(A)** and SCM versus GYS **(B)** fecal metabolites. Volcano plot of CON versus SCM **(C)** and SCM versus GYS **(D)** differential metabolites. **(E)** Venn diagram outlining the shared differentially abundant metabolites in CON versus SCM and SCM versus GYS. **(F)** Heatmap showing differences in shared metabolites among the CON, SCM, and GYS groups.

Subsequently, we conducted an untargeted metabolomics analysis of fecal samples from the four groups. In order to elucidate the metabolite distinctions among the groups, an orthogonal partial least squares discriminant analysis (OPLS-DA) was conducted on fecal samples. As demonstrated in Figures 4A,B, the CON group can be distinguished from the SCM group, and the SCM group from the GYS group. This finding indicates that there have been substantial changes in the intestinal metabolites of dairy cows with subclinical mastitis treated with GYS. Following the filtration and optimization steps, a total of 4,467 metabolites were identified from 24 fecal samples. A total of 35 metabolites exhibited significant differences between the CON and SCM groups, including 34 metabolites that were up-regulated and 1 metabolite that was down-regulated in the SCM group (|log2FC| ≥ 2, VIP > 1 and *p* < 0.05) (Figure 4C). A total of 41 metabolites exhibited significant differences between the SCM and GYS groups, including 14 metabolites that were up-regulated and 27 metabolites that were down-regulated in the GYS group (Figure 4D). We identified 24 shared metabolites in the CON, SCM and GYS groups (Figure 4E), including Efavirenz, Benfuresate, Alangicine, Ethyl icosapentate, Asn-Ala-Leu-Ala-His, Lys-Ile-Glu, Diclofenac sodium, Docosahexaenoic acid methyl ester, Nomifensine maleate, 1-Oleoyl-2-myristoyl-sn-glycero-3-phosphocholine, 7alpha-Hydroxy-3-oxochol-4-en-24-oic acid, Oleoyltaurine, Lys-Pro-Lys, Fenaminosulf, Gentian violet, Cavipetin C, His-Arg-Lys-Glu, Arg-Gln-Arg, 5-O-beta-D-mycaminosyl-20-oxotylonolide, Phe-Asn-Leu, Malyngamide J, Ala-Thr-Ile-Lys, 3,6,9,12,15,18,21-Heptaoxatricosane-21,23-diol, Arg-Thr-Ala-Arg, and interestingly, all these metabolites were upregulated in the SCM group and significantly decreased after GYS treatment (Figure 4F).

**Figure 5:**
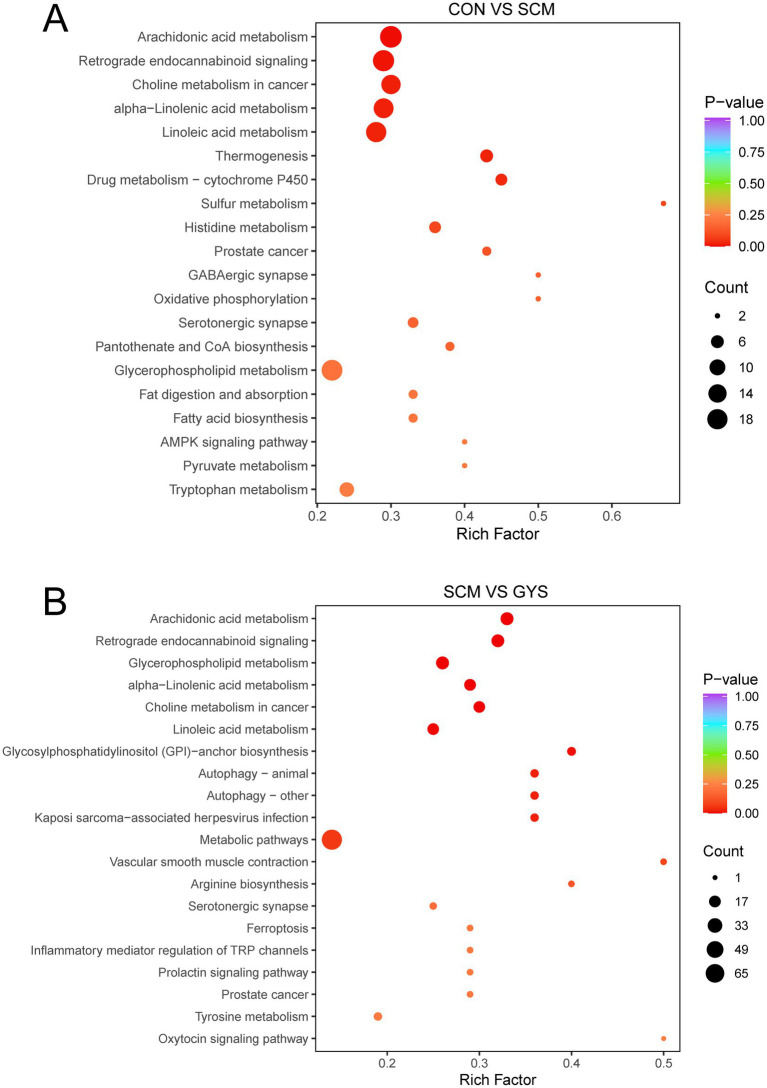
KEGG enrichment pathways analysis. Metabolic pathway analysis conducted with the differentially abundant fecal metabolites in the CON versus SCM **(A)** and SCM versus GYS **(B)** comparisons.

Metabolic pathways were annotated based on different metabolites using KEGG database. Arachidonic acid metabolism, retrograde endocannabinoid signaling, choline metabolism in cancer, alpha-Linolenic acid metabolism, linoleic acid metabolism, and glycerophospholipid metabolism were the pathways that were significantly different between the CON and SCM groups (Figure 5A). In the metabolome of SCM and GYS groups arachidonic acid metabolism, retrograde endocannabinoid signaling, glycerophospholipid metabolism, alpha-Linolenic acid metabolism, choline metabolism in cancer, linoleic acid metabolism, glycosylphosphatidylinositol (GPI)-anchor biosynthesis, etc. were significantly different pathways (Figure 5B). By comparison, the co-enriched pathways were found to be Arachidonic acid metabolism, Retrograde endocannabinoid signaling, Glycerophospholipid metabolism, alpha-Linolenic acid metabolism, Choline metabolism in cancer and Linoleic acid metabolism.

### 3.6 Significant correlations between different fecal microbiota and milk components, and between metabolites and fecal microbiota

**Figure 6:**
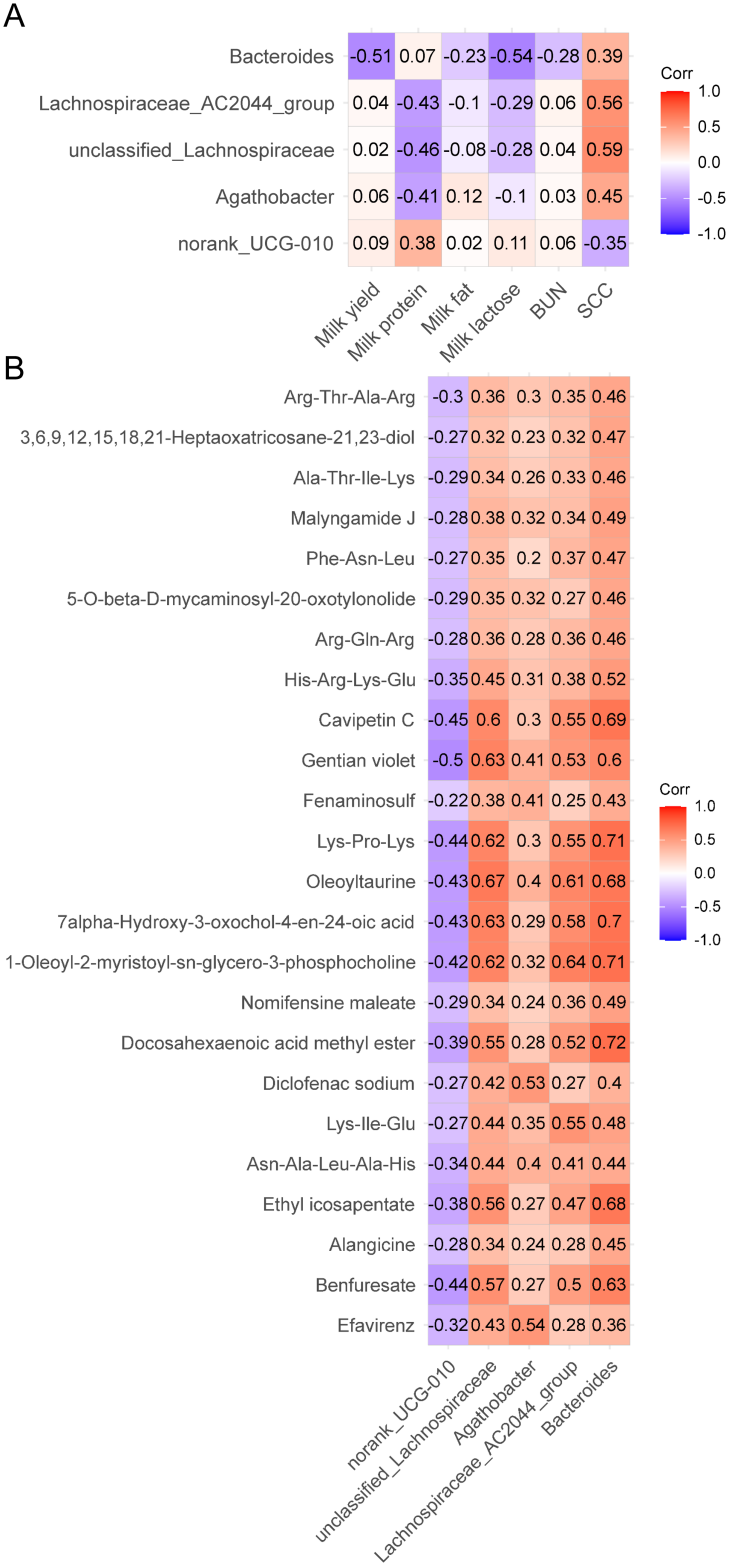
Analysis of the correlation between significant different fecal microbiota and milk compositions **(A)** and differential metabolites **(B)**. Each lattice represents a Pearson correlation coefficient between a bacterium and a metabolite or milk compositions. Red represents a positive correlation, while blue represents a negative correlation.

First, we investigated the correlation between differential microbiota and lactation performance. Bacteroides was negatively correlated with milk yield (*p =* 0.003) and milk lactose (*p =* 0.001). *Norank_UCG-010* (*p =* 0.030) was positively correlated with milk protein, by contrast, *Unclassified_Lachnospiraceae* (*p =* 0.009), *Agathobacter* (*p =* 0.020) and *Lachnospiraceae_AC2044_group* (*p =* 0.013) were negatively correlated with milk protein. *Unclassified_Lachnospiraceae* (*p* < 0.001), *Agathobacter* (*p =* 0.010), *Bacteroides* (*p =* 0.026) and *Lachnospiraceae_AC2044_group* (*p* < 0.001) were positively correlated with milk SCC, but *norank_UCG-010* (*p =* 0.049) was negatively correlated with milk SCC (Figure 6A).

For metabolites, Benfuresate (*p =* 0.031), 1-Oleoyl-2-myristoyl-sn-glycero-3-phosphocholine (*p =* 0.039), 7alpha-Hydroxy-3-oxochol-4-en-24-oic acid (*p =* 0.034), Oleoyltaurine (*p =* 0.036), Lys-Pro-Lys (*p =* 0.030), Gentian violet (*p =* 0.013) and Cavipetin C (*p =* 0.029) were negatively correlated with *norank_UCG-010*. Efavirenz (*p =* 0.007), Diclofenac sodium (*p =* 0.008), Fenaminosulf (*p =* 0.044) and Gentian violet (*p =* 0.048) were positively correlated with *Agathobacter*. Efavirenz (*p =* 0.036), Benfuresate (*p =* 0.003), Ethyl icosapentate (*p =* 0.005), Asn-Ala-Leu-Ala-His (*p =* 0.030), Lys-Ile-Glu (*p =* 0.032), Diclofenac sodium (*p =* 0.041), Docosahexaenoic acid methyl ester (*p =* 0.006), 1-Oleoyl-2-myristoyl-sn-glycero-3-phosphocholine (*p =* 0.001), 7alpha-Hydroxy-3-oxochol-4-en-24-oic acid (*p* < 0.001), Oleoyltaurine (*p* < 0.001), Lys-Pro-Lys (*p =* 0.001), Gentian violet (*p* < 0.001), Cavipetin C (*p =* 0.002) and His-Arg-Lys-Glu (*p =* 0.029) were positively correlated with *unclassified_Lachnospiraceae*. Benfuresate (*p =* 0.012), Ethyl icosapentate (*p =* 0.020), Asn-Ala-Leu-Ala-His (*p =* 0.044), Lys-Ile-Glu (*p =* 0.005), Docosahexaenoic acid methyl ester (*p =* 0.009), 1-Oleoyl-2-myristoyl-sn-glycero-3-phosphocholine (*p* < 0.001), 7alpha-Hydroxy-3-oxochol-4-en-24-oic acid (*p =* 0.003), Oleoyltaurine (*p =* 0.002), Lys-Pro-Lys (*p =* 0.006), Gentian violet (*p =* 0.007) and Cavipetin C (*p =* 0.005) were positively correlated with *Lachnospiraceae_AC2044_group*. All differential metabolites except Efavirenz and Diclofenac sodium were positively correlated with *Bacteroides* (*p* < 0.050) (Figure 6B).

## 4 Discussion

Although the udders and milk of cows with SCM do not show significant changes in appearance, the milk yield and composition can be severely compromised. Antibiotics are still the drug of choice for the treatment of mastitis, but the misuse of antibiotics in animal husbandry has led to a series of problems such as bacterial resistance, environmental pollution and harm to human health (20–22). Therefore, the appropriate addition of botanicals appears to be critical to the lactation performance of cows. Yue Wang et al. reported that supplementation with inulin improved lactation performance and milk quality in cows with SCM (23). Furthermore, the addition of 200 g/day of dandelion has been shown to enhance milk production and lactose levels in dairy cows (12). In herbal formulas, the interplay among components can exhibit agonistic, compatible, or antagonistic effects, resulting in the modulation of multiple signaling pathways in various organs, thereby contributing to disease alleviation (24). In this study, we observed a significant decrease in lactose levels in cows with SCM, which may be attributed to the inflammation-induced increase in blood-milk barrier permeability. GYS treatment led to a substantial reduction in serum LPS levels, suggesting that intestinal permeability was ameliorated, which leads to elevated lactose levels (25). Furthermore, the increase in milk production due to GYS can be attributed to the cure of SCM. Although CEF treatment group reduced milk SCC, the milk yield was still significantly lower than that of the CON group. Antibiotics destroy beneficial microbial communities in the cow’s gut, affecting fiber breakdown and nutrient absorption, leading to impaired energy metabolism, which in turn reduces the supply of raw materials needed for milk synthesis.

Mastitis in dairy cows is frequently accompanied by systemic low-grade inflammation, and increased inflammatory factors are important in compromising the integrity of the blood-milk barrier (26, 27). It has been demonstrated that TNF-α has the capacity to compromise the integrity of the intestinal barrier by inducing the shedding of intestinal epithelial cells (28). IL-6 activates the JAK–STAT signaling pathway, resulting in an increase in the permeability of the blood–brain barrier (29). LPS can enter the bloodstream from the intestinal and cause a range of inflammatory responses that disrupt barrier integrity by reducing the expression of tight junction proteins (30). The present study demonstrated that serum inflammatory factors were significantly elevated in cows with SCM. Furthermore, levels of IL-2, IL-4, IL-8, IL-10, and TNF-α were significantly reduced after GYS treatment, suggesting that GYS ameliorated the inflammatory response induced by SCM. The reduction in LPS suggests that the integrity of the intestinal and blood-milk barriers is also improved, but direct evidence is still needed for further proof. In addition, consistent with the findings of most studies, GYS has been shown to improve antioxidant activity by increasing SOD and decreasing MDA (12, 31–33). In the CEF group, we also found that the levels of IL-4 and TNF-α were reduced, which may be related to the alteration of intestinal microbiota by antibiotics (34). Surprisingly, TP, ALB, and GLB levels did not change during SCM. This result is consistent with the study by Yue et al. (23). Elevated immunoglobulin levels due to prolonged low-grade inflammation may be offset by lowering of other types of globulins, resulting in no change in GLB levels. Several studies have shown the inhibitory effect of IL-6 on albumin synthesis (35–37). In the present study, there was also no significant change in IL-6 levels, which may explain the lack of change in ALB. In conclusion, the addition of GYS was found to be more effective in alleviating the inflammatory response in SCM cows.

Alpha diversity analysis and PCOA revealed that the diversity of the microbiota in the CEF group was significantly lower and the structure of the microbiota was significantly altered compared to the other three groups. This is consistent with the results of most studies that antibiotics may have contributed to the imbalance of the intestinal microbiota (38, 39). A large body of evidence suggests that there are significant differences in the structure and function of the rumen and intestinal microbiota between healthy cows and cows with mastitis (19, 40). In this study, we found that *Bacteroides* was significantly elevated in the SCM group compared to the CON and GYS groups. It has been reported that *Bacteroides* can synthesize putrescine and spermidine *in vitro* and *in vivo* (41). Polyamines through c-Myc modulating intestinal epithelial barrier function (42). In addition, intestinal-derived enterotoxigenic *Bacteroides fragilis* caused mammary epithelial hyperplasia, suggesting that *Bacteroides* disrupt the mammary barrier (43). In addition, *Lachnospiraceae*, *Prevotellaceae*, and *Bifidobacteriaceae*, which were elevated in the SCM group, are fiber-degrading microbiota that produce short-chain fatty acids (SCFAs) by fermenting plant fibers and other carbohydrates. This may lead to an abnormal elevation of SCFAs, which in turn leads to a decrease in pH in the rumen, thus triggering subacute rumen acidosis (SARA) (44). It has been found that SARA is often accompanied by a systemic inflammatory response and mastitis (45). Although *Lachnospiraceae* and *Agathobacter* are generally considered to be beneficial microbiota, the present study found both to be in high abundance in the feces of cows with SCM (46, 47). Caijun et al. also found that *Lachnospiraceae* and *Agathobacter* were present in high levels in the intestinal tracts of cows with mastitis and mice with rumen microbial transplants (40, 48). Furthermore, these levels were found to be positively correlated with inflammatory factors. *Norank_UCG-010* belongs to the group of rumenococci that are involved in the breakdown of starch and fiber in ruminants and contribute to the further digestion of feed in the intestinal (49). *UCG-010* is negatively correlated with inflammatory factors (IL-6, IL-12, and IL-17) (50). Furthermore, an increase in *Blautia* abundance was observed in the GYS group. *Blautia* is defined as having potential probiotic properties, and it plays a role in reducing inflammation (51). GYS is characterized by its richness in flavonoids, including quercetin, luteolin, and kaempferol (18). These flavonoids can be metabolized by *Blautia* to form bioactive substances, which may account for the enrichment of *Blautia* (52). Therefore, the findings suggest that GYS can provide relief from SCM by influencing the inflammation-related microbiota restoring a healthy community structure. Among them, *UCG-010* has potential application in the treatment of mastitis.

Several correlated metabolites (e. g., Efavirenz, Diclofenac sodium, Gentian violet) are synthetic drugs or chemicals not expected to be present in untreated dairy cows. Environmental contamination or dietary sources have not been confirmed, and their correlation with specific taxa may result from mis-annotation or database error. It is evident that alterations in the composition of intestinal microbes give rise to concomitant changes in metabolites. We saw that 1-Oleoyl-2-myristoyl-sn-glycero-3-phosphocholine was more abundant in the SCM group and less abundant in the GYS group. 1-Oleoyl-2-myristoyl-sn-glycero-3-phosphocholine belongs to the group called phosphatidylcholines, which have been shown to be converted by intestinal bacteria into a harmful substance called trimethylamine-N-oxide (TMAO) (53). This activates a pathway in the body called the NF-κB pathway, which triggers the production of cytokines (54). Furthermore, in the present study 7alpha-Hydroxy-3-oxochol-4-en-24-oic acid was found to be elevated in the SCM group compared to the CON and GYS groups. 7alpha-Hydroxy-3-oxochol-4-en-24-oic acid belongs to the group of 3-oxo-Δ4 bile acids (55). And the accumulation of 3-oxo-Δ4 bile acids replaces primary bile acid conjugates, resulting in a decrease in secondary bile acid synthesis (56). Deoxycholic acid-mediated activation of the G protein-coupled receptor reduces inflammation by inhibiting the NF-κB and NLRP3 pathways and improves the integrity of the blood-milk barrier, thereby reducing mastitis (57, 58). In the present study, phosphatidylcholine and 3-oxo-Δ4 bile acids were positively correlated with *Lachnospiraceae* and *Bacteroides*, negatively correlated with *UCG-010*, suggesting that they may be regulated by microbiota, which requires further study.

On the other hand, intestinal metabolic pathways were significantly altered after GYS treatment, and these changes were mainly related to lipid metabolism. We found that Arachidonic acid metabolism and Choline metabolism were downregulated in the GYS group. Arachidonic acid amplifies inflammatory signals to promote the production of leukocytes, pro-inflammatory cytokines and immune cells to fight and eliminate pathogens (59). Moreover, we found that phosphatidylcholine was elevated in the SCM group and that phospholipase could induce the release of arachidonic acid from phosphatidylcholine via TNF-α binding to its receptor (60). Endogenous cannabinoids are also a source of arachidonic acid (61). Although linoleic acid is an essential fatty acid, it can be elongated and desaturated to arachidonic acid, which in turn leads to inflammation (62). Phosphatidylcholine is synthesized from choline via the CDP-choline pathway (63), and elevated phosphatidylcholine implies increased cellular uptake of choline, which is often accompanied by an inflammatory response (64–66). Therefore, GYS may alleviate SCM by affecting Arachidonic acid metabolism and Choline metabolism, with Phosphatidylcholine being the key factor. Based on correlation analyses, it was hypothesized that *UCG-010*, *Lachnospiraceae_AC2044_group* and *Bacteroides* may influence secondary bile acid synthesis as well as choline metabolism via 3-oxo-Δ4 bile acids and phosphatidylcholine.

## 5 Conclusion

The present study demonstrated that GYS enhanced milk yield, and improved lactation performance in cows with SCM. GYS reduced serum inflammatory cytokines, LPS and MDA levels, elevated SOD levels, and reduced microbiota associated with inflammation and intestinal barriers in dairy cows with SCM, thus, GYS attenuated inflammation and oxidative stress in serum. Furthermore, we observed substantial alterations in arachidonic acid metabolism and choline metabolism, with the metabolite phosphatidylcholine being reduced by GYS. This suggests that GYS may alleviate inflammation through this pathway. In conclusion, the use of GYS could be considered as an effective alternative therapeutic strategy to antibiotics, with the potential to reduce the risk of systemic inflammation in cows with SCM.

## Data Availability

The original contributions presented in the study are publicly available. This data can be found here: NCBI BioProject, accession PRJNA1268138.

## Footnotes

^1^ https://cutadapt.readthedocs.io/en/stable/

^2^ https://cme.h-its.org/exelixis/web/software/pear/

^3^ https://prinseq.sourceforge.net/

^4^ https://drive5.com/uparse/

^5^ https://www.arb-silva.de/

^6^ https://rdp.cme.msu.edu/

^7^ https://mothur.org/

^8^ https://www.r-project.org/

^9^ https://bitbucket.org/biobakery/biobakery/wiki/lefse

^10^ https://hmdb.ca/

^11^ https://www.kegg.jp/kegg/pathway.html
