# Supplementary material for: Integrated multi-omics analysis reveals that Gongying San ameliorates subclinical mastitis by modulating intestinal microbiota and metabolites in dairy cows
Source: Front Vet Sci. 2025 Jun 9;12:1589900. doi: 10.3389/fvets.2025.1589900 (PMC12183554; doi:10.3389/fvets.2025.1589900)
Supplement: Supplementary file 1 [file Table_1.DOCX]

**Table S1** Ingredients and chemical composition of basal diet

| **Ingredients** | **%** | **Chemical composition** | **%** |
| --- | --- | --- | --- |
| Corn | 40 | DM | 90 |
| Soya meal | 28.9 | CP | 21.6 |
| Rumen fed soybean meal | 5 | EE | 7.3 |
| DDGS | 12 | NDF | 13.5 |
| Glucose | 2 | Ca | 1.12 |
| Fatty powder | 2.5 | P | 0.6 |
| XP | 0.9 | ME (MJ/kg) | 13.2 |
| Slow-release protein | 0.5 |  |  |
| Mildew removing agent | 0.1 |  |  |
| Premix | 5.6 |  |  |
| Sodium bicarbonate | 2.5 |  |  |
| Total | 100 |  |  |

DDGS, distillers dried grains with solubles; XP, yeast culture; DM, dry matter; CP, crude prodein; EE, ether extract; NDF, neutral detergent fiber; ME, metabolic energy; Premix, including (per kg of diets) 400 KIU of vitamin A, 160 KIU of vitamin D_3_, ≥ 2, 200 IU of vitamin E, 600 mg of Cu, 3, 400 mg of Zn, 3, 400 mg of Mn, 12 mg of Se, 60 mg of I, 40 mg of Co, ≥ 20 % of Ca, ≥ 4 % of Mg and ≥ 6 % of Na.


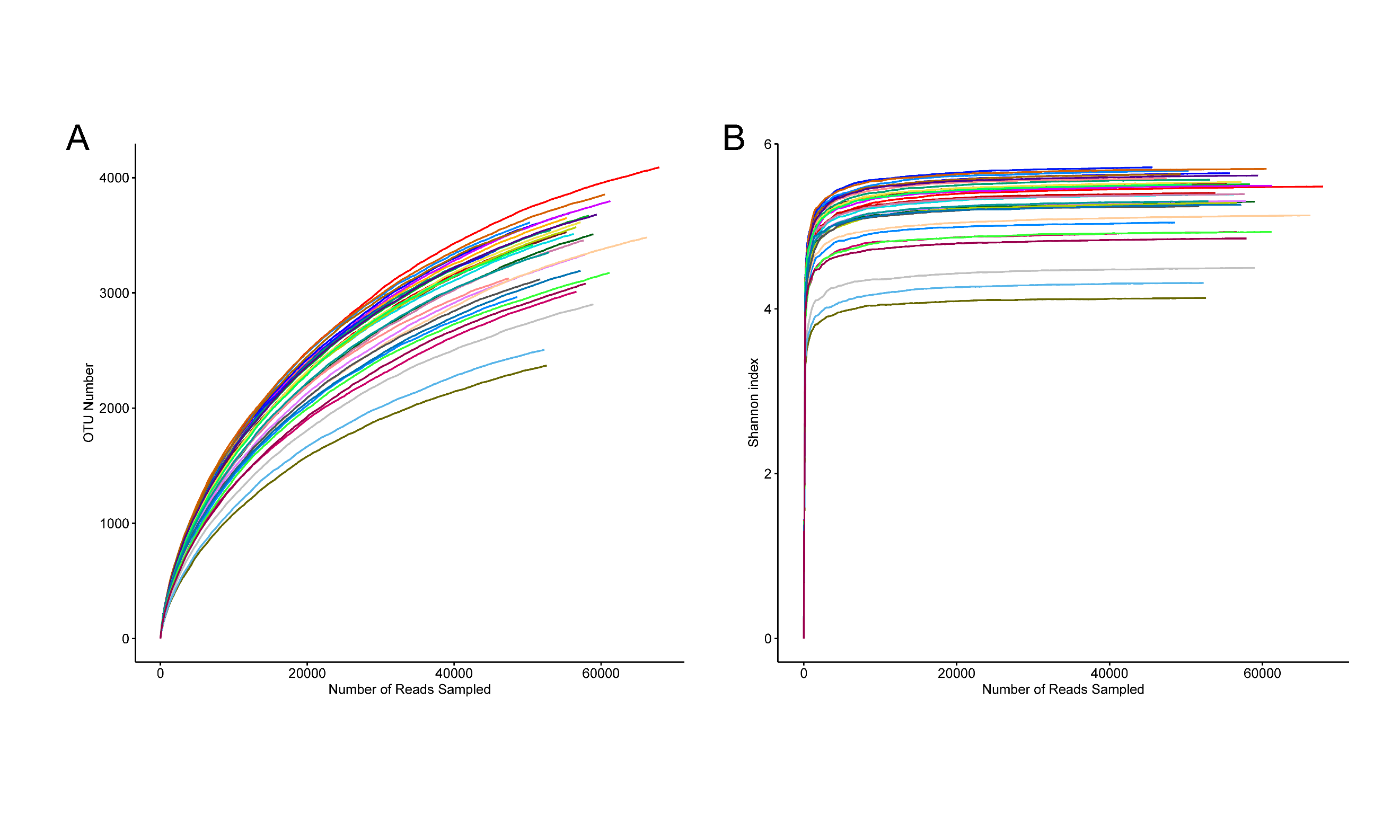


**Fig. S1** Rarefaction curves based on (A) OUT number and (B) Shannon indexes. The abscissa is the number of randomly selected sequences in the sample, the ordinate is the corresponding Alpha index, and each curve represents one sample.
